# New High‐*T*_c_ Charge‐Transfer Multiferroicity in the Quasi‐2D Antiferromagnet CrSbS_3_

**DOI:** 10.1002/advs.202523579

**Published:** 2026-04-02

**Authors:** Hung‐Cheng Wu, Yu‐Hsin Liao, Chih‐Chung Tsai, Pei‐Ying Yang, Yu‐Hao Chang, Yu‐Hui Liang, D. Chandrasekhar Kakarla, Ajay Tiwari, C. Dhanasekhar, Chin‐Wei Wang, Daisuke Okuyama, Chia‐Nung Kuo, Chin‐Shan Lue, Tzu‐Hsuan Weng, Kung‐Hsuan Lin, Jau‐Wern Chiou, Chi‐Hung Lee, Hung‐Duen Yang

**Affiliations:** ^1^ Department of Physics National Sun Yat‐Sen University Kaohsiung Taiwan; ^2^ National Synchrotron Radiation Research Center Hsinchu Taiwan; ^3^ Institute of Materials Structure Science High Energy Accelerator Research Organization (KEK) Tsukuba Ibaraki Japan; ^4^ Department of Physics National Cheng Kung University Tainan Taiwan; ^5^ Taiwan Consortium of Emergent Crystalline Materials (TCECM) National Science and Technology Council Taipei Taiwan; ^6^ Institute of Physics Academia Sinica Taipei Taiwan; ^7^ Department of Applied Physics National University of Kaohsiung Kaohsiung Taiwan; ^8^ Department of Applied Physics Tunghai University Taichung Taiwan

**Keywords:** charge transfer, high‐T_c_ type‐II multiferroic, inversion‐symmetry breaking, magnetic space group Pnm'a

## Abstract

Low‐dimensional magnets, particularly 2D systems, offer a rich platform for realizing unconventional multiferroic mechanisms, especially when multiple polarization channels coexist. In the quasi‐2D antiferromagnet CrSbS_3_, which crystallizes in the centrosymmetric orthorhombic space group *Pnma* and orders magnetically at *T*_N_ ≈ 90 K, two independent polarization mechanisms are uncovered. A weak spontaneous polarization (P_1_) emerges near *T*_E_ ≈ *T*_N_ at zero field, driven by partial Cr charge transfer. Synchrotron XRD refinements reveal anomalies in bond valences and average Cr─S bond length, XAS detects subtle modifications in local electronic states, and Raman spectroscopy uncovers anomalous phonon softening, indicating a charge‐transfer‐driven reconstruction of the lattice and phonon. In addition, second‐harmonic generation (SHG) measurements exhibit a clear enhancement below *T*_E_, directly confirming inversion‐symmetry breaking and supporting the emergence of the spontaneous polarization P_1_. Further, neutron diffraction reveals a C‐type antiferromagnetic structure described by the magnetic space group *Pnm'a*, which breaks inversion symmetry and induces an additional polarization (P_2_) under applied magnetic fields. Critical exponent analysis (*β* ≈ 0.23(2)) highlights quasi‐2D magnetism. These results establish CrSbS_3_ as a rare high‐*T*_c_/*T*_N_ (∼90 K) type‐II multiferroic, where charge, spin, lattice, and phonon degrees of freedom cooperatively produce robust ferroic responses in a 2D framework.

## 1 Introduction

Low‐dimensional magnetic materials have emerged as fertile systems for discovering new correlated and emergent phases [1, 2, 3, 4] because reducing dimensionality strengthens spin‐charge‐lattice interactions and enables responses rarely seen in bulk compounds. Breakthroughs in van der Waals magnets such as Cr_2_Ge_2_Te_6_ and CrI_3_, and Fe_3_GeTe_2_ have demonstrated stable 2D ferromagnetism [1, 2, 3, 4], while layered transition‐metal chalcogenides, including centrosymmetric MnPS_3_ and non‐centrosymmetric Cu_1‐x_Mn_1+y_SiTe_3_, have shown symmetry‐allowed linear magnetoelectricity and spin‐driven ferroelectricity [5, 6, 7]. These advances illustrate how dimensional reduction promotes unconventional magnetic states, enhances fluctuations, and amplifies cross‐coupling among multiple degrees of freedom, thereby motivating accelerating efforts to identify 2D and quasi‐2D multiferroics [8, 9, 10, 11].

Despite advances in both layered and bulk spin‐driven multiferroics, most known systems still order below ∼60 K [6, 7, 11, 12, 13, 14, 15], greatly limiting technological utility. Achieving high‐*T*_c_ multiferroicity remains a central challenge. Low‐dimensional platforms—where reduced symmetry and flexible lattice environments strengthen spin‐lattice interactions—offer a promising route toward realizing electric polarization at elevated temperatures.

Within this context, the MPnCh_3_ (M = 3d transition metal; Pn = P/Sb; Ch = S/Se) family has emerged as a tunable platform in which delicate competition among charge localization, exchange geometry, and lattice flexibility generates diverse electronic and magnetic phases [5, 6, 16, 17, 18, 19, 20, 21, 22, 23, 24, 25]. For example, CrSbSe_3_ crystallizes in the orthorhombic structure but exhibits quasi‐1D ferromagnetism near 70 K with anisotropic magnetocaloric effects and undergoes a pressure‐driven transition to superconductivity [21], demonstrating remarkable sensitivity to lattice tuning. By contrast, isostructural CrSbS_3_ displays antiferromagnetic ordering at ∼90 K accompanied by sequential Cr‐charge transfer [20], hinting at coupled electronic and magnetic instabilities. However, its ferroic responses remain unknown, leaving open whether charge redistribution and magnetism cooperate to generate polarization.

Beyond dimensionality, symmetry provides a rigorous framework for discovering ferroics [14, 26, 27, 28, 29]. In centrosymmetric materials, ferroelectricity can only appear when symmetry is broken by magnetic or structural ordering, while magnetic symmetry determines whether spin‐driven polarization at zero magnetic field [29], linear magnetoelectric effect under applied fields [14, 27], anomalous Hall effects, or piezomagnetism are allowed [26, 28]. Thus, magnetic‐space‐group analysis enables symmetry‐guided prediction of ferroicity before experiments.

The *Pnma* space group represents an instructive prototype for symmetry‐enabled ferroics [20, 26, 27]. Although structurally centrosymmetric, different magnetic configurations can yield magnetic subgroups that lift inversion symmetry and permit polarization. Well‐established examples include LiFePO_4_ (*Pnma'*), a linear magnetoelectric effect [27, 30], and YbNiSn (*Pn'm'a*), which hosts weak ferromagnetism and symmetry‐controlled anomalous Hall switching [26]. CrSbSe_3_ (*Pnm'a'*), while maintaining the same structural symmetry, displays quasi‐1D ferromagnetism and evolves into a superconducting state under pressure [21], illustrating remarkable symmetry tunability of its electronic ground states. These systems collectively underscore that *Pnma* lattices provide fertile ground for symmetry‐tunable ferroic responses.

CrSbS_3_ crystallizes in *Pnma*, which forbids ferroelectricity unless inversion symmetry is broken by magnetic order [20]. For the magnetic propagation vector *k* = (0, 0, 0), representation analysis yields eight symmetry‐allowed magnetic configurations, four of which—*Pn'ma*, *Pnm'a*, *Pnma'*, and *Pn'm'a'*—permit magnetoelectric coupling. Prior neutron diffraction proposed the *Pnm'a* ground state [20], placing CrSbS_3_ precisely in a symmetry channel capable of generating magnetically induced polarization. Combined with its remarkably high Néel temperature (∼90 K) within the low‐dimensional MPnCh_3_ family, CrSbS_3_ emerges as a compelling candidate for high*‐T*_c_ multiferroicity.

CrSbS_3_ also exhibits highly anisotropic thermal expansion near *T*_N_, characterized by negative thermal expansion (NTE) along the *c* axis, positive thermal expansion (PTE) along *a*, and nearly zero thermal expansion (ZTE) along *b*. [20] Theoretically, NTE driven by spin‐lattice coupling has been proposed in honeycomb antiferromagnets composed of edge‐sharing MX_6_ octahedra, where two competing nearest‐neighbor exchange channels—direct 180° M‐M antiferromagnetic exchange and ∼90° M‐X‐M ferromagnetic superexchange—respond oppositely to bond‐length variations [31, 32]. Although CrSbS_3_ does not form a honeycomb lattice, its zigzag Cr network hosts the same exchange motif, i.e., one direct Cr‐Cr AFM path and two nearly 90° Cr‐S‐Cr FM pathways. This structural similarity provides an ideal platform to test this mechanism [31, 32]. The emergence of NTE in CrSbS_3,_ therefore, reflects strong magnetoelastic coupling and demonstrates a microscopic link between lattice distortion, magnetic exchange competition, and electronic degrees of freedom in this quasi‐2D system.

Here, we demonstrate that CrSbS_3_ is a rare quasi‐2D high‐*T*_c_ (∼90 K) type‐II multiferroic hosting two independent polarization responses: a weak zero‐field spontaneous polarization driven by partial Cr charge transfer, and a magnetic‐field‐induced polarization arising from inversion‐symmetry breaking in the *Pnm'a* magnetic phase. We further establish that the NTE originates from competing magnetic exchange pathways consistent with a spin‐lattice‐coupled mechanism. These results identify CrSbS_3_ as a unique member of the MPnCh_3_ family and demonstrate a symmetry‐guided design pathway toward realizing high‐*T*_c_ type‐II multiferroics in low‐dimensional systems.

## 2 Result and Discussion

### 2.1 Structural Characterization and Quasi‐2D Cr Sublattice

**FIGURE 1:**
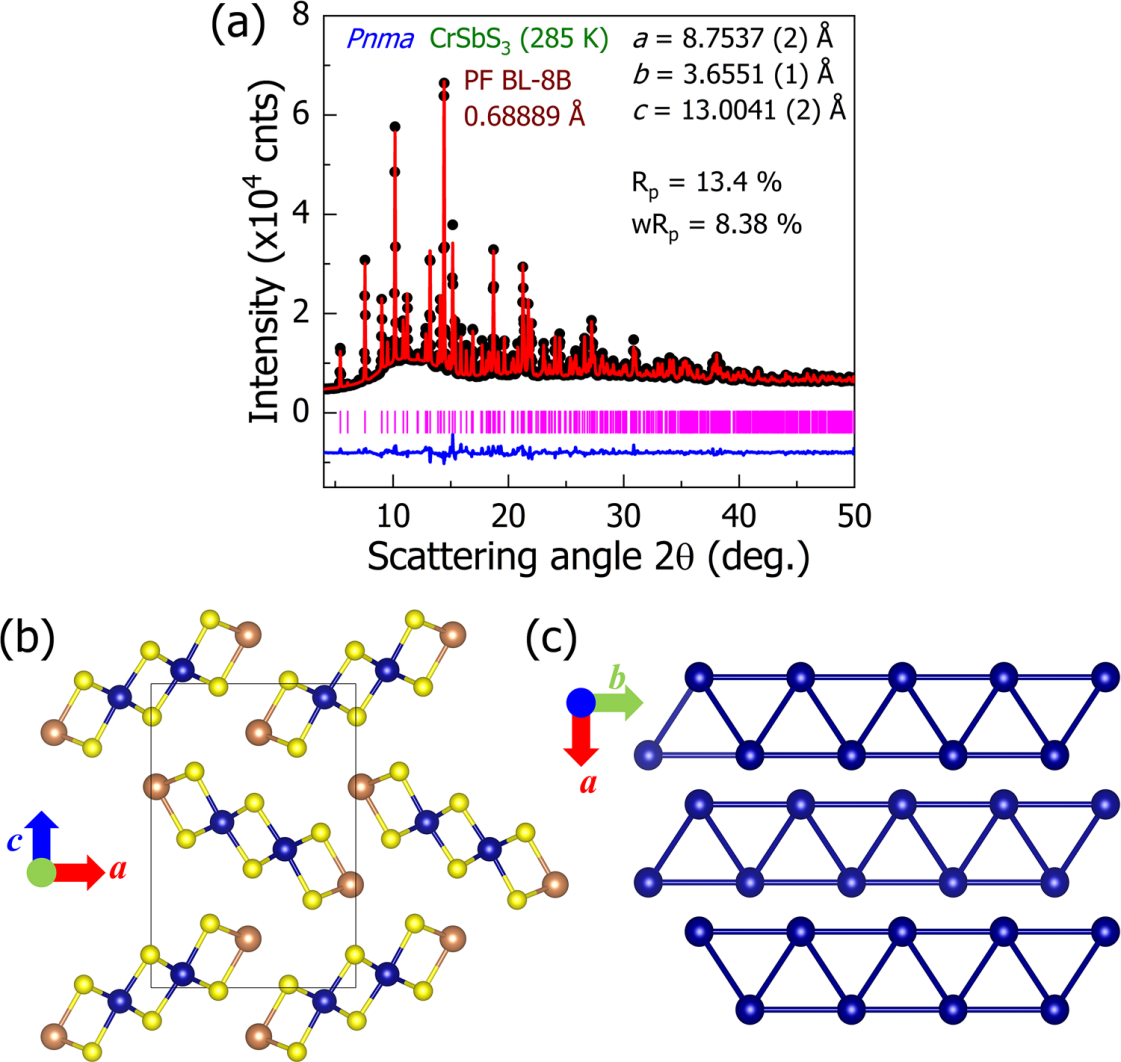
(a) High‐resolution synchrotron powder XRD pattern of CrSbS_3_ recorded at 285 K (λ = 0.68889 Å, PF BL‐8B) together with Rietveld refinement (red line), demonstrating the single‐phase nature of the sample. (b) The refined crystal structure of CrSbS_3_ can be visualized as a network of edge‐sharing CrS_6_ octahedra interconnected with Sb atoms that stabilize the lattice. (c) When the Cr sublattice is viewed along the *c* axis, zigzag chains arranged in layers become evident, revealing a quasi‐2D framework that may give rise to intriguing magnetic properties. Crystal structure visualizations were generated using the VESTA software [42].

High‐resolution synchrotron X‐ray diffraction (SXRD) was employed to verify the phase purity and crystallographic quality of CrSbS_3_. As shown in Figure 1a, all X‐ray reflections can be indexed using the orthorhombic space group *Pnma* [20], and Rietveld refinement yields excellent agreement between the measured and calculated profiles, confirming the single‐phase nature of the sample. The refined lattice parameters (*a* = 8.7537(2) Å, *b* = 3.6551(1) Å, *c* = 13.0041(2) Å) and low refinement residuals (R_p_ = 13.4%, wR_p_ = 8.38%) further verify the structural reliability, summarized in Table 1. Following the structural validation, we examined the atomic arrangement of CrSbS_3_ (Figure 1b) in the *ac* plane. The structure consists of edge‐sharing CrS_6_ octahedra interconnected by Sb‐S units, forming extended Cr‐S networks in the *ac* plane. Although the global symmetry remains centrosymmetric (*Pnma*), visualizing only the Cr sublattice along the *c* axis (Figure 1c) reveals zigzag chains stacked into layers within the *ab* plane, establishing a quasi‐2D magnetic framework. Such a layered zigzag geometry naturally enhances in‐plane exchange interactions while suppressing interlayer coupling, providing a structural foundation for the quasi‐2D magnetism and symmetry‐breaking phenomena discussed below.

**TABLE 1:** Refined lattice parameters, atomic positions, number of X‐ray reflections, fitting parameters, and Rietveld reliability indicators for CrSbS_3_ at 285 K, obtained from synchrotron X‐ray diffraction measurements at PF BL‐8B. The χ^2^ value is slightly larger than unity because the high counting statistics of synchrotron data reduce the expected residual (R_exp_). Therefore, R_p_ and wR_p_ provide a more appropriate measure of refinement quality.

CrSbS_3_ Orthorhombic *Pnma*, (No. 62), *T* = 285 K (PF BL‐8B) *a* = 8.7537(2) Å, *b* = 3.6551(1) Å, *c* = 13.0041(2) Å) *α* = 90^ο^, *β* = 90^ο^, *γ* = 90^ο^				

Atom	Site	*x*	*y*	*z*
Cr	4*c*	0.1546 (5)	1/4	0.0459 (4)
Sb	4*c*	0.9741 (2)	3/4	0.3399 (1)
S1	4*c*	1.0010 (9)	3/4	0.1054 (4)
S2	4*c*	0.7963 (7)	1/4	0.2889 (6)
S3	4*c*	0.8230 (7)	3/4	0.5096 (5)

Number of X‐ray reflections: 2556.

Number of fitting parameters: 51.

R_p_ = 13.4%, wR_p_ = 8.38%, R_exp_ = 1.17%, χ^2^ = 51.5.

### 2.2 Magnetization Characterization, Neutron Diffraction, Magnetic Structure Determination, and Order Parameter Analysis

**FIGURE 2:**
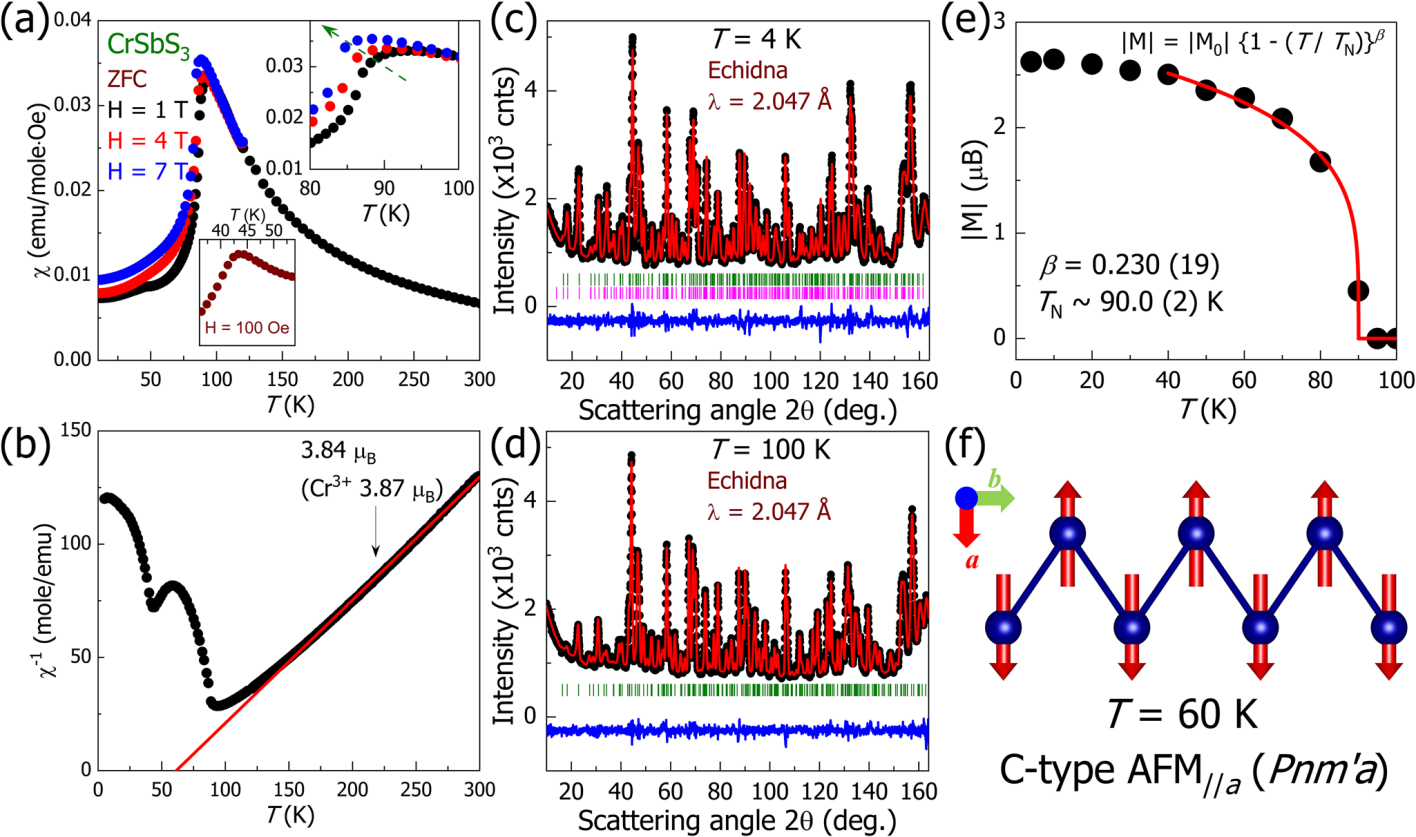
(a) Temperature‐dependent magnetic susceptibility of CrSbS_3_ under different applied magnetic fields shows two magnetic anomalies: the main transition at *T*_N_ ≈ 90 K and a secondary feature at *T* ^*^ ≈ 45 K. While *T* ^*^ is quickly suppressed by external fields, *T*_N_ persists and shifts slightly toward lower temperatures, underscoring its robustness. The upper inset focuses on the region around *T*_N_, showing its shift under applied fields, while the lower inset depicts the weaker anomaly at *T* ^*^ that appears under H = 100 Oe. (b) Curie‐Weiss law analysis of the inverse susceptibility yields an effective magnetic moment of 3.84 µ_B_/Cr^3+^, in excellent agreement with the expected spin‐only value of Cr^3+^ (3.87 µ_B_), confirming the trivalent nature of Cr ions in CrSbS_3_. (c) Neutron powder diffraction pattern of CrSbS_3_ collected at 4 K (λ = 2.047 Å, Echidna) with Rietveld refinement (red line). The green tick marks indicate nuclear reflections, while the pink ones represent magnetic contributions. (d) The diffraction pattern measured at 100 K, above *T*_N_, contains only nuclear reflections, confirming that the additional peaks at 4 K are magnetic in origin. (e) Temperature dependence of the ordered moment fitted with a critical power law, yielding *β* = 0.230(19). This value, close to the expected quasi‐2D limit (*β* ≈ 0.25), demonstrates that the magnetic transition is dominated by in‐plane interactions with weak interlayer coupling, consistent with the layered zigzag Cr network. (f) The refined magnetic structure at 60 K corresponds to a C‐type antiferromagnetic order with spins collinearly aligned along the *a* axis, described by the magnetic space group *Pnm'a*. The breaking of inversion symmetry in this magnetic phase provides a natural explanation for the emergence of magnetically induced polarization (P_2_). Magnetic structure visualizations were generated using the VESTA software [42].

To elucidate the magnetic properties of CrSbS_3_, temperature‐dependent magnetic susceptibility measurements were performed under various magnetic fields (Figure 2a). At a low magnetic field (100 Oe), two anomalies are clearly observed: a primary transition at *T*_N_ ≈ 90 K and a weaker secondary feature at *T* ^*^ ≈ 43.5 K, as highlighted in the lower inset. Upon increasing the magnetic field, the signature at *T* ^*^ rapidly diminishes, demonstrating its field‐sensitive and fragile nature. In contrast, the anomaly at *T*_N_ persists and shifts slightly to lower temperatures under applied fields (upper inset), indicating a robust long‐range magnetic ordering with moderate field tunability. Given the pronounced stability of the *T*_N_ transition and its correlation with neutron, dielectric, pyroelectric, structural, second‐harmonic generation (SHG), and Raman signatures presented below, our discussion focuses on the physics associated with *T*_N_. The Curie‐Weiss analysis of the high‐temperature susceptibility (Figure 2b) yields an effective moment of µ_eff_ = 3.84 µ_B_/Cr, in excellent agreement with the expected spin‐only value for Cr^3+^ (3.87 µ_B_), confirming the trivalent state and localized‐moment nature of chromium ions in CrSbS_3_.

Neutron powder diffraction was employed to resolve the magnetic ground state of CrSbS_3_. Figure 2c shows the diffraction pattern collected at 4 K. Rietveld refinement, incorporating both nuclear and magnetic contributions, successfully reproduces all observed peaks. The additional reflections appearing at low temperature (pink tick marks) are absent in the dataset acquired at 100 K (Figure 2d), confirming their magnetic origin and establishing the onset of long‐range antiferromagnetic order below *T*_N_.

To determine the magnetic ground state, symmetry‐allowed magnetic configurations were examined using magnetic representation analysis. For the Cr ions occupying the 4*c* Wyckoff position in the orthorhombic space group *Pnma*, the magnetic representation decomposes into eight one‐dimensional irreducible representations (IR1‐IR8). The basis vectors corresponding to each irreducible representation, generated using the BasIreps module in the FullProf Suite together with their associated magnetic space groups, are summarized in Table 2. Evaluation of the magnetic reflection conditions for all symmetry‐allowed models reveals that only IR5 is compatible with the experimentally observed magnetic Bragg peaks. Although symmetry allows both *a*‐ and *c*‐axis components for IR5, refinement of the *c* component yielded negligible values with large uncertainties. Constraining the moment along the *a* axis significantly improved the refinement stability, indicating that the ordered moment is predominantly collinear along *a*. Refinement of this representation yields a C‐type antiferromagnetic configuration with spins oriented along the *a* axis (Figure 2f). This magnetic configuration corresponds to the magnetic space group *Pnm'a* [20], which is non‐centrosymmetric although the parent *Pnma* structure is centrosymmetric. The loss of inversion symmetry induced purely by magnetic ordering indicates that magnetically driven polarization is symmetry‐allowed below *T*_N_, providing a strong motivation to search for magnetoelectric responses at elevated temperatures.

**TABLE 2:** Symmetry‐allowed magnetic irreducible representations for the Cr atom occupying the 4*c* Wyckoff site of the *Pnma* space group, evaluated for the commensurate propagation vector *k* = (0, 0, 0). The basis vectors associated with each representation are listed for the four crystallographically distinct Cr positions. IR5 (highlighted in bold) is the only representation consistent with the observed magnetic Bragg reflections and corresponds to a C‐type antiferromagnetic arrangement with magnetic moments aligned along the *a* axis. Notably, C‐type antiferromagnetic orders within the *Pnma* structural family are symmetry‐compatible with linear magnetoelectric coupling [40, 41]. Compared with those systems [40, 41], CrSbS_3_ exhibits a substantially higher transition temperature (≈ 90 K), highlighting its advantage as a high‐*T*_c_ magnetoelectric platform.

IR	(*x*, ¼, *z*)	(‐*x* + ½, ¾, *z* + ½)	(‐*x* + 1, ¾, ‐*z* + 1)	(*x* + ½, ¼, ‐*z* + ½)	Magnetic category	Magnetic space group
IR1	(0, *m_b_*, 0)	(0, ‐*m_b_*, 0)	(0, *m_b_*, 0)	(0, ‐*m_b_*, 0)	G‐AFM_||_ *_b_*	*Pnma*
IR2	(0, *m_b_*, 0)	(0, *m_b_*, 0)	(0, ‐*m_b_*, 0)	(0, ‐*m_b_*, 0)	C‐AFM_||_ *_b_*	*Pn'ma*
IR3	(0, *m_b_*, 0)	(0, ‐*m_b_*, 0)	(0, ‐*m_b_*, 0)	(0, *m_b_*, 0)	A‐AFM_||_ *_b_*	*Pnma'*
IR4	(0, *m_b_*, 0)	(0, *m_b_*, 0)	(0, *m_b_*, 0)	(0, *m_b_*, 0)	FM_||_ *_b_*	*Pn'ma'*
**IR5**	**(** ***m**_**a**_* **,** **0** **,** ***m**_**c**_* **)**	**(** ***m**_**a**_* **,** **0** **,** **‐** ***m**_**c**_* **)**	**(** **‐** ***m**_**a**_* **,** **0** **,** **‐** ***m**_**c**_* **)**	**(** **‐** ***m**_**a**_* **,** **0** **,** ***m**_**c**_* **)**	**C‐AFM**_**||**_ *_**a**_* **A‐AFM**_**||**_ *_**c**_*	***Pnm'a***
IR6	(*m_a_*, 0, *m_c_*)	(‐*m_a_*, 0, *m_c_*)	(‐*m_a_*, 0, ‐*m_c_*)	(*m_a_*, 0, ‐*m_c_*)	A‐AFM_||_ *_a_* C‐AFM_||_ *_c_*	*Pn'm'a'*
IR7	(*m_a_*, 0, *m_c_*)	(‐*m_a_*, 0, *m_c_*)	(*m_a_*, 0, *m_c_*)	(‐*m_a_*, 0, *m_c_*)	G‐AFM_||_ *_a_* FM_||_ *_c_*	*Pn'm'a*
IR8	(*m_a_*, 0, *m_c_*)	(*m_a_*, 0, ‐*m_c_*)	(*m_a_*, 0, *m_c_*)	(*m_a_*, 0, ‐*m_c_*)	FM_||_ *_a_* G‐AFM_||_ *_c_*	*Pnm'a'*

The temperature evolution of the ordered Cr moment (Figure 2e) follows a critical power‐law behavior |*M*| = *M*_0_ (1−*T*/*T*_N_)*^β^*, yielding *β* = 0.230(19) and *T*_N_ ≈ 90.0 K. The extracted critical exponent closely matches the expected range for quasi‐2D antiferromagnet (*β* ≈ 0.23–0.25) [33], consistent with the layered zigzag Cr framework established structurally. Together, the magnetization, magnetic structure refinement, and critical‐exponent analysis firmly establish that CrSbS_3_ undergoes a quasi‐2D magnetic transition that intrinsically breaks inversion symmetry. This symmetry condition (*Pnm'a*) provides a natural platform on which high‐*T*_c_ magnetoelectric and multiferroic responses may emerge, motivating the investigations presented in the following sections.

### 2.3 Dielectric, Pyroelectric Responses, and Polarization

**FIGURE 3:**
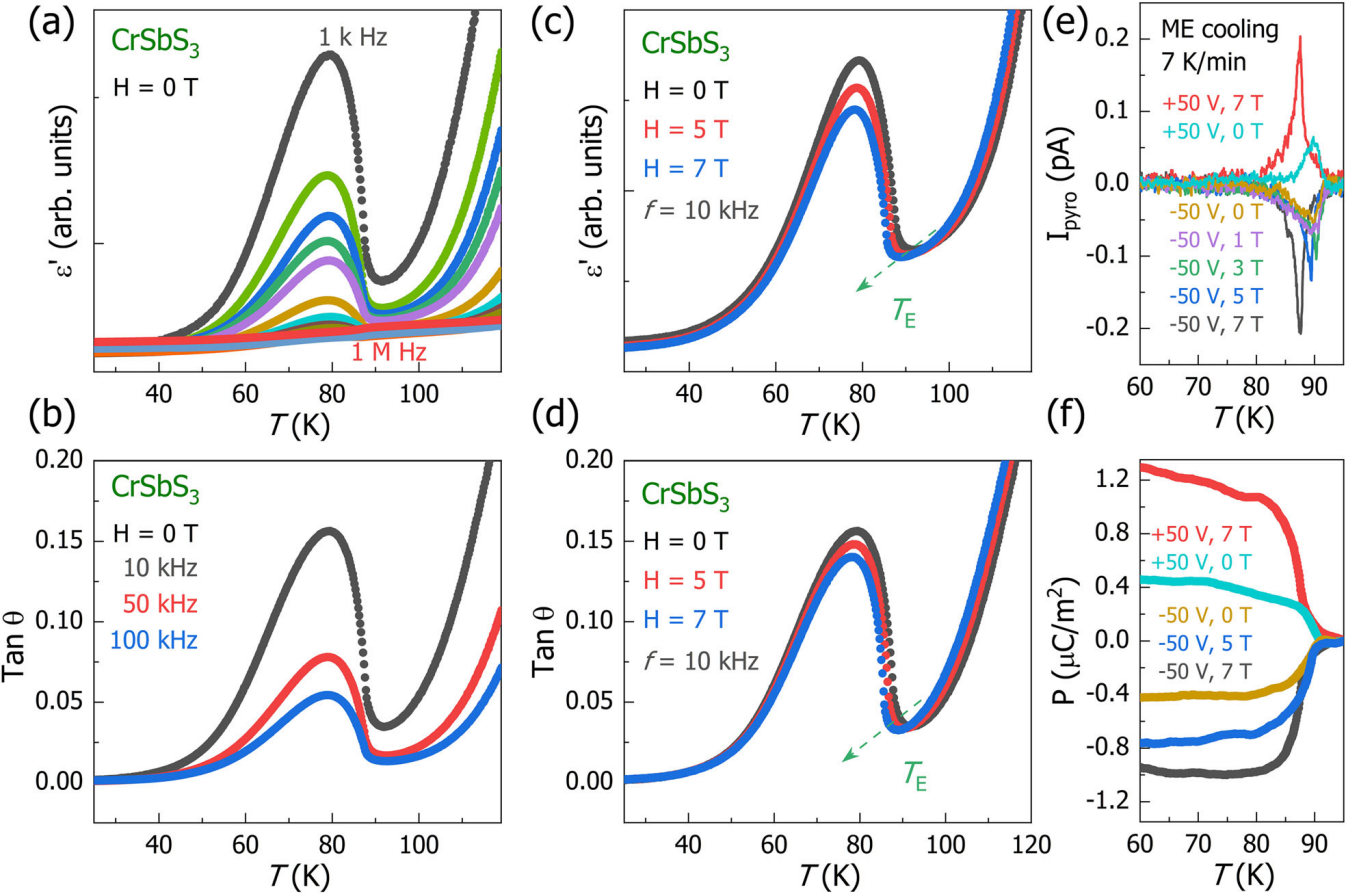
(a) Temperature‐dependent dielectric constant of CrSbS_3_ under zero field at frequencies ranging from 1 kHz to 1 MHz, showing a sharp anomaly at *T*_E_ ≈ 90 K whose position is frequency‐independent, indicative of a long‐range electrical order. (b) Temperature‐dependent dielectric loss (tan θ) of CrSbS_3_ measured under zero field at 10, 50, and 100 kHz. (c) Under an applied magnetic field (5 and 7 T, *f* = 10 kHz), the anomaly at *T*_E_ shifts slightly to lower temperatures, consistent with the field dependence observed in magnetization. (d) Temperature‐dependent dielectric loss (tan θ) of CrSbS_3_ measured at 10 kHz under magnetic fields of 0, 5, and 7 T. (e, f) Pyrocurrent measurements performed after magnetoelectric (ME) cooling to ensure a single‐domain state reveal the emergence of a spontaneous polarization P_1_ at zero field, with an additional magnetic‐field‐induced contribution P_2_ that grows with increasing field. The integrated polarization curves confirm that both P_1_ and P_2_ can be reversed by switching the poling electric field, demonstrating their ferroelectric nature. P_1_ and the associated inversion‐symmetry breaking below *T*_E_.

The dielectric response of CrSbS_3_ was first examined under zero magnetic field across a wide frequency range (1 kHz to 1 MHz), as shown in Figure 3a. A pronounced anomaly appears near *T*_E_ ≈ 90 K, where the dielectric constant sharply increases upon cooling. Importantly, the *T*_E_ transition temperature shows negligible frequency dependence, signaling a long‐range electrical ordering rather than a relaxor‐type response. To probe magnetoelectric coupling, dielectric measurements were performed under magnetic fields up to 7 T (Figure 3c, *f* = 10 kHz). The anomaly at *T*_E_ gradually shifts to lower temperature with increasing field, mirroring the trend observed in magnetization. This field‐induced shift confirms a strong coupling between magnetic and dielectric degrees of freedom. The emergence of polarization near *T*_E_ was further investigated through pyroelectric current measurements after magnetoelectric (ME) cooling to ensure a single‐domain state (Figure 3e). A weak spontaneous pyroelectric signal appears at zero magnetic field (P_1_), demonstrating the onset of a polar state without an external magnetic field. Under finite magnetic fields, the pyroelectric peak grows in magnitude, indicating an additional field‐induced polarization component (P_2_). Integration of the pyroelectric current yields the temperature‐dependent polarization (Figure 3f), where both the zero‐field and field‐induced components reverse upon switching the poling electric field, confirming their ferroelectric nature. Together, these results reveal that CrSbS_3_ hosts both spontaneous and field‐induced polarization at the same transition temperature near 90 K, establishing a coupled ferroic response that motivates deeper investigation of its microscopic origin in subsequent sections.

Importantly, the appearance of the field‐induced component P_2_ is fully consistent with the magnetic symmetry analysis in Section 2.2. The C‐type antiferromagnetic order belongs to the noncentrosymmetric magnetic space group *Pnm′a*, meaning that inversion symmetry is broken only in the magnetically ordered state. Such magnetic symmetry reduction intrinsically allows a linear magnetoelectric response, naturally explaining the emergence of the field‐induced polarization P_2_.

In contrast, the spontaneous zero‐field polarization P_1_—which develops even without an applied magnetic field—cannot be accounted for by magnetic symmetry alone and therefore requires an independent microscopic mechanism.

### 2.4 Temperature‐Dependent Bond‐Valence (BV), and X‐Ray Absorption Spectroscopy (XAS)

**FIGURE 4:**
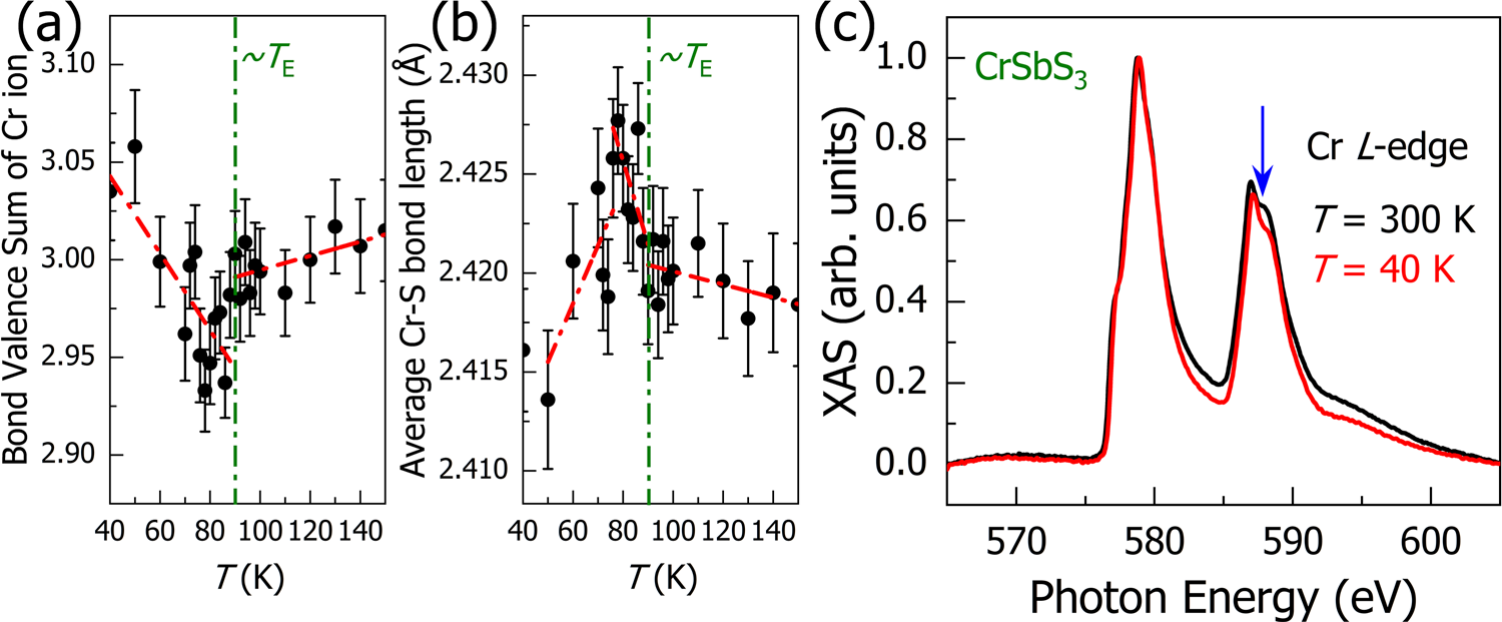
Temperature‐dependent (a) bond valence and (b) average Cr─S bond length obtained from synchrotron XRD refinements exhibit clear anomalies near *T*_E_ ≈ 90 K. The bond valence and bond length variations suggest a finite charge transfer of Cr ions, which provides the microscopic origin of the spontaneous polarization (P_1_). (c) Cr *L*‐edge XAS spectra measured at 300 and 40 K show subtle spectral changes, corroborating partial charge transfer.

To uncover the microscopic origin of the spontaneous zero‐field polarization P_1_, the bond‐valence of Cr and average Cr─S bond length were extracted from the SXRD refinements (Figure 4a,b). To clarify the relationship between the bond valence sum and the average bond length shown in Figure 4, these quantities are interpreted within the bond‐valence framework. The bond valence sum shown in Figure 4a reflects the valence‐sum rule, which requires that the sum of individual bond valences around an atom equals its atomic valence [34]. As observed here, the bond valence sum remains close to the nominal valence over the measured temperature range. Under this constraint, structural adjustments are accommodated through a redistribution of individual bond lengths. According to the distortion theorem of the bond‐valence model, changes in the distribution of bond lengths under a fixed bond valence sum can lead to variations in the average bond length [34]. Consistent with this description, the average Cr─S bond length shown in Figure 4b deviates from a simple monotonic thermal behavior and exhibits a turning point near *T*_E_. This behavior reflects a modification in bond‐length redistribution within the bond‐valence description. Both quantities deviate from a smooth thermal trend near *T*_E_, indicating a subtle yet finite redistribution of Cr electronic charge. Such behavior is consistent with a partial charge‐transfer process across the transition [35, 36], offering a natural microscopic basis for the emergence of zero‐field polarization P_1_. The magnitude of this charge redistribution is modest, in line with the relatively small P_1_ value observed in pyroelectric measurements. Further support comes from Cr *L*‐edge X‐ray absorption spectroscopy (Figure 4c), which exhibits a slight but reproducible modification of the *L*_2_ spectrum between 300 and 40 K. This spectral evolution reflects a measurable change in the local electronic configuration of Cr ions. Together, these structural and spectroscopic results demonstrate that charge transfer accompanies the transition at *T*_E_, providing the essential mechanism for the spontaneous polarization P_1_.

### 2.5 Temperature‐Dependent Lattice Parameters, and Second Harmonic Generation (SHG)

**FIGURE 5:**
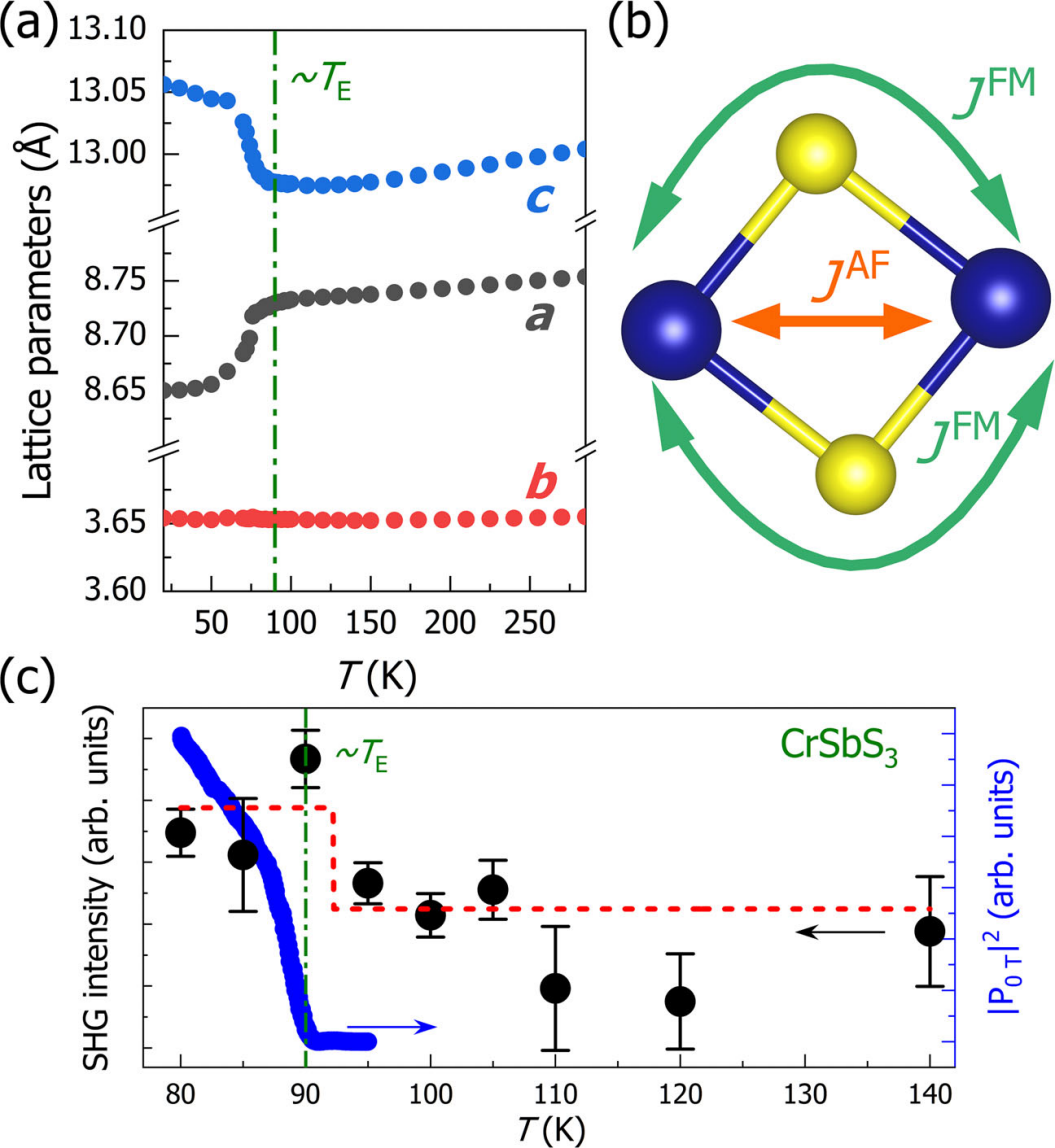
(a) Temperature‐dependent lattice parameters obtained from synchrotron XRD refinements exhibit clear anomalies near *T*_E_ ≈ 90 K, evidencing strong magnetoelastic coupling. (b) A spin‐lattice model derived from neutron diffraction refinements illustrates the competition between one direct antiferromagnetic exchange (*J*_AF_​) and two nearly 90° ferromagnetic superexchange (*J*_FM_), whose delicate balance accounts for the observed negative thermal expansion along the *c*‐axis. Local crystal structure visualizations were generated using the VESTA software [42]. (c) Temperature dependence of the second‐harmonic generation (SHG) intensity (black circles), together with the squared zero‐field polarization |P_0 T_|^2^ extracted from pyroelectric measurements (blue curve). The parallel evolution of the two signals supports the emergence of the spontaneous polarization and the associated inversion‐symmetry breaking below *T*_E_.

Temperature‐dependent SXRD refinements reveal pronounced lattice anomalies at *T*_E_ (Figure 5a). Upon cooling, the *c* axis undergoes a clear crossover from positive thermal expansion (PTE) to negative thermal expansion (NTE), whereas the *a* and *b* axes display PTE and nearly zero thermal expansion (ZTE), respectively. This highly anisotropic behavior signals a lattice instability coincident with the onset of magnetic, dielectric, and polarization anomalies, highlighting strong magnetoelastic coupling in CrSbS_3_.

The origin of the NTE along *c* can be explained by the proposed spin‐lattice mechanism illustrated in Figure 5b [31, 32]. Within the Cr zigzag network, a direct Cr‐Cr interaction favors antiferromagnetic exchange *J*_AFM_, while two nearly 90° Cr‐S‐Cr pathways contribute ferromagnetic superexchange *J*_FM_. The delicate competition between *J*_AFM_ and *J*_FM_ drives a magnetoelastic response that contracts the lattice upon cooling, fully consistent with both neutron‐refined C‐type AFM order and the experimentally observed NTE behavior. Taken together, these results establish that the anomaly at *T*_E_ arises from a coupled charge‐lattice‐spin reconstruction linking the emergence of P_1_ with the magnetoelastic response.

To directly verify the inversion‐symmetry breaking associated with P_1_, temperature‐dependent SHG measurements were performed on pelletized CrSbS_3_. As shown in Figure 5c, the SHG intensity shows a distinct enhancement below *T*_E_. Since CrSbS_3_ exhibits the centrosymmetric *Pnma* space group above *T*_E_, SHG is mainly contributed from higher‐order contributions such as magnetic dipole and electric quadrupole. The enhancement below *T*_E_ supports the argument of inversion symmetry breaking, where the electric dipole additionally contributes to SHG. When compared with the squared zero‐field polarization, the onset of SHG provides direct optical evidence for the emergence of the spontaneous polarization P_1_.

### 2.6 Raman Spectra and Spin‐Phonon Coupling

**FIGURE 6:**
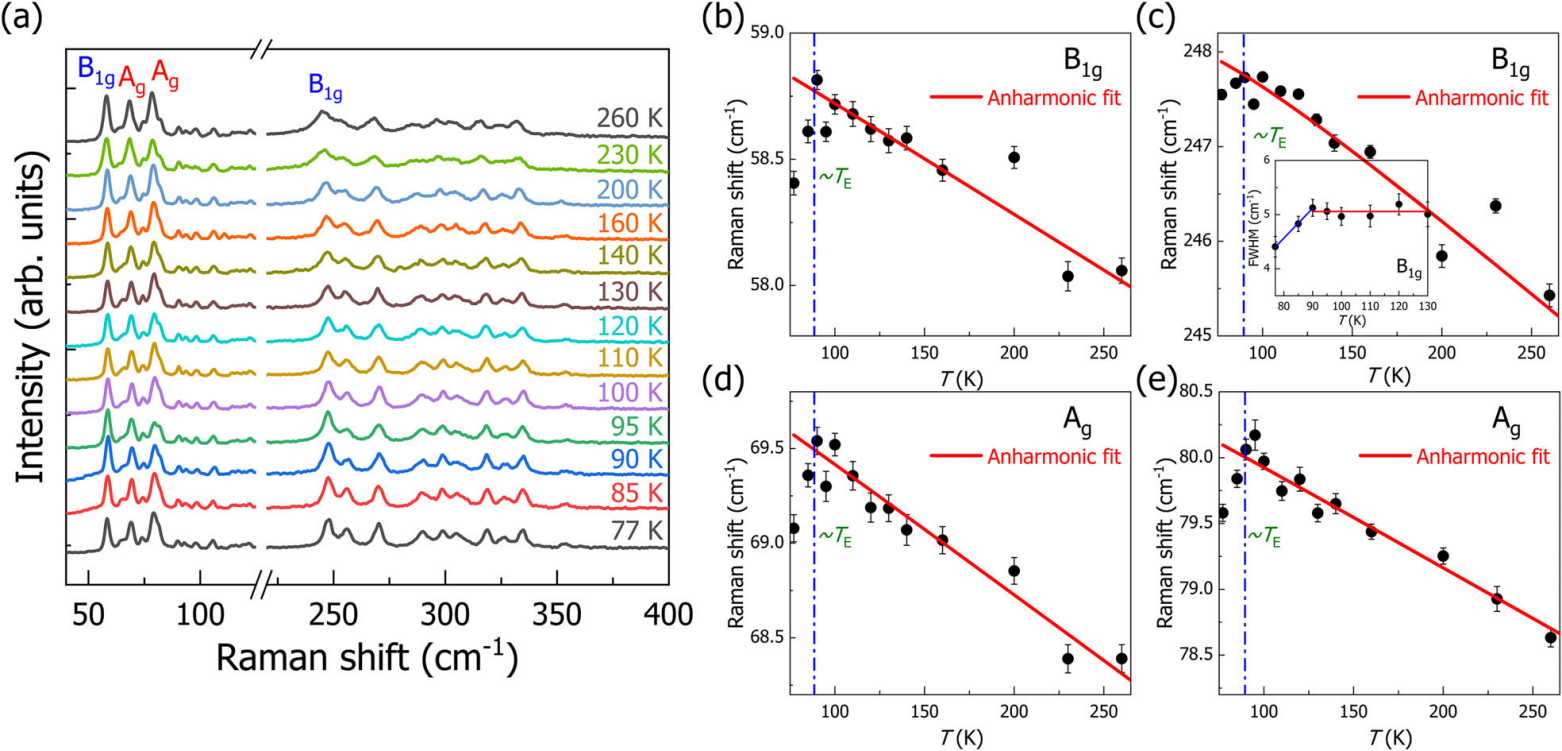
(a) Temperature‐dependent Raman spectra of CrSbS_3_ measured from 77 to 260 K. Temperature‐dependent Raman shifts of representative phonon modes of CrSbS_3_ fitted with the anharmonic decay model (red lines): (b, c) B_1g_ vibrational modes parallel to the chain direction and (e, d) A_g_ vibrational modes perpendicular to the chain direction. Above *T*_E_, the overall frequency evolution follows the expected anharmonic hardening upon cooling. Below *T*_E_, each of the four Raman shifts shows a clear deviation from the anharmonic trend, indicative of anomalous phonon softening. These results point to pronounced spin‐phonon coupling emerging near *T*_E_, in agreement with the proposed spin‐lattice model for negative thermal expansion. The inset of panel (c) displays the temperature dependence of the phonon linewidth in B_1g_ mode, which exhibits a clear narrowing below *T*_E_, further supporting the presence of spin‐phonon coupling [43].

To elucidate the lattice dynamics across *T*_E_ in CrSbS_3_, temperature‐dependent Raman spectroscopy was performed, and four representative phonon branches were monitored (Figure 6). Two B_1g_ modes are dominated by vibrations along the Cr zigzag chains (along *b* axis), while the two A_g_ modes correspond to lattice motions perpendicular to the chains (along *a* axis) [20]. These modes lie within the *ab* plane, consistent with the quasi‐2D zigzag Cr network illustrated in Figure 1c. Tracking both chain‐parallel and chain‐perpendicular vibrations therefore enables us to directly examine phonon behavior associated with the anisotropic quasi‐2D lattice framework in CrSbS_3_.

In the absence of structural instability or coupling to spin or electronic degrees of freedom, phonon modes are expected to follow a conventional anharmonic hardening upon cooling. This behavior can be described by the standard anharmonic model: $\omega_{anh}\left(T\right)=\omega_{0}\;-C\left(1+\frac{2}{e^{\frac{\hbar \omega_{0}}{k_{B}T}}-1}\right)$ [37], where ω_0_ denotes the intrinsic phonon frequency at 0 K, *C* quantifies phonon‐phonon interactions, ℏ is the reduced Planck constant, and *k_B_* is the Boltzmann constant. As shown in Figure 6b–e, above *T*_E_, all four phonon branches follow the expected anharmonic trend and harden upon cooling, consistent with purely phonon‐phonon interactions. However, below *T*_E_, all modes exhibit a pronounced softening, providing direct spectroscopic evidence for the emergence of spin‐phonon coupling. Importantly, both the chain‐parallel and chain‐perpendicular vibrations display comparable softening, demonstrating that the spin‐phonon coupling is not limited to a specific vibrational direction but instead reflects a cooperative lattice response within the quasi‐2D Cr network.

By integrating the spin‐driven negative thermal expansion established in Section 2.5 with the Raman evidence for spin‐phonon coupling below *T*_E_, a unified picture emerges in which magnetic order strongly modulates the lattice dynamics in CrSbS_3_. This spin‐mediated lattice response parallels that observed in the layered van der Waals magnet CrBr_3_ below its magnetic transition, where phonon anomalies accompany magnetically induced lattice distortion [38]. Taken together with magnetic, dielectric, polarization, BV/XAS, lattice‐parameter, and SHG results, these findings demonstrate the emergence of a cooperative spin‐charge‐lattice coupling below *T*_E_ = *T*_N_, giving rise to the high*‐T*_c_ multiferroic phenomena in this quasi‐2D van der Waals antiferromagnet. Such intertwined degrees of freedom suggest that external pressure or chemical substitution offer promising pathways to manipulate the coupled order parameters and further enhance the transition temperature in this correlated system.

## 3 Conclusion

In summary, our systematic investigation of the quasi‐2D antiferromagnet CrSbS_3_ reveals a rare high‐*T*_c_ (>77 K) type‐II multiferroic, where two independent polarization mechanisms emerge within the same material. At *T* ≈ 90 K, a weak spontaneous polarization (P_1_) develops under zero magnetic field, driven by partial charge transfer of Cr ions. This zero‐field polar state is further supported by the emergence of SHG below *T*_E_, confirming inversion‐symmetry breaking. In contrast, an additional polarization (P_2_) appears only under magnetic fields, arising from the inversion‐symmetry breaking of the *Pnm'a* magnetic space group. This dual origin reflects the cooperative action of charge, spin, lattice, and phonon degrees of freedom. The latter is directly evidenced by Raman spectroscopy, which detects anomalous phonon softening and highlights pronounced spin‐phonon coupling near *T*_E_. These intertwined responses also manifest in the quasi‐2D critical behavior and the anomalous negative thermal expansion along the *c*‐axis. Rather than being separate effects, they form a unified framework where charge transfer stabilizes high‐*T*_c_ multiferroicity, while spin‐lattice‐phonon coupling governs its microscopic character. Through this comprehensive approach, the case of CrSbS_3_ demonstrates how low‐dimensional chalcogenides can host unconventional high‐*T*_c_ ferroic states, pointing toward promising directions for designing next‐generation high‐*T*_c_ multiferroic materials and magnetoelectric functionalities.

## 4 Experimental Section

### 4.1 Materials Synthesis

Polycrystalline CrSbS_3_ was prepared via a solid‐state reaction method. Stoichiometric amounts of Cr (99.9%), Sb (99.99%), and S (99.99%) powders in a molar ratio of 1:1:3 were mixed and thoroughly ground inside an Ar‐filled glovebox. The homogeneous mixture was sealed in an evacuated quartz tube and heated to 650°C for 48 h. After furnace cooling to room temperature, the product was reground, resealed, and annealed under the same conditions to ensure phase purity.

### 4.2 Synchrotron X‐Ray Powder Diffraction

Temperature‐dependent synchrotron X‐ray powder diffraction (SXRD) measurements were performed at beamline BL‐8B of the Photon Factory (KEK, Japan) using a monochromatic beam of 18 keV (λ = 0.68889 Å). Polycrystalline CrSbS_3_ powder was loaded into a 0.2 mm glass capillary and sealed with Stycast epoxy in a He atmosphere (∼40% He) to avoid air exposure and ensure efficient thermal exchange at low temperatures. Diffraction patterns were collected from 20 to 285 K, and all data could be indexed within the orthorhombic *Pnma* symmetry without any detectable structural transition across the measured range. Rietveld refinement was carried out using the FullProf Suite [39], from which lattice parameters and atomic coordinates were obtained at each temperature. Based on the refined structural parameters, the bond‐valence sum (BVS) of Cr and the average Cr─S bond length were further extracted to probe charge redistribution and local structural evolution across the transition.

### 4.3 Neutron Diffraction

Neutron powder diffraction measurements were performed on the Echidna high‐resolution diffractometer at ANSTO (Australia) with a neutron wavelength of λ = 2.047 Å. Polycrystalline CrSbS_3_ powder was sealed in a vanadium can, and diffraction patterns were collected from 4 to 100 K. The magnetic Bragg peaks emerging below the transition temperature were refined using the FullProf Suite [39]. The temperature‐dependent ordered moment extracted from sequential refinements was fitted using a power‐law model to obtain the critical exponent. The magnetic structure was determined to be a C‐type antiferromagnet with spins aligned along *a* axis, corresponding to the magnetic space group *Pnm'a*.

### 4.4 Magnetization

DC magnetization measurements were performed using a SQUID magnetometer (MPMS‐XL 7, Quantum Design). Approximately 10.6 mg of polycrystalline CrSbS_3_ powder was sealed in a non‐magnetic capsule. Temperature‐dependent magnetization was recorded under zero‐field‐cooled (ZFC) conditions, with selected applied magnetic fields up to 7 T.

### 4.5 Dielectric and Pyrocurrent

Dielectric properties were measured using an Agilent E4980A LCR meter with an AC excitation voltage of 10 V and frequencies ranging from 1 kHz to 1 MHz. Pyroelectric current was recorded with a Keithley 6517B electrometer. A custom‐designed probe compatible with the MPMS‐XL7 system was employed to enable in situ dielectric and pyroelectric measurements under simultaneous temperature and magnetic‐field control. Polycrystalline CrSbS_3_ was configured in a parallel‐plate capacitor geometry by applying silver paste to the top and bottom surfaces as electrodes, with the electric field oriented perpendicular to the applied magnetic field. For pyroelectric measurements, the sample was cooled under simultaneous magnetic and electric fields (magnetoelectric cooling) to ensure a single‐domain state. During warming at a constant temperature‐ramp rate, the pyroelectric current was continuously recorded and integrated to obtain polarization.

### 4.6 X‐Ray Absorption Spectroscopy

Temperature‐dependent X‐ray absorption spectroscopy (XAS) measurements were performed at the Cr *L*‐edge using beamline BL45A2 at the Taiwan Photon Source (NSRRC). The spectra were collected in total‐electron‐yield (TEY) mode with the sample mounted in an open‐helium flow cryostat.

### 4.7 Raman Spectroscopy

Temperature‐dependent Raman spectroscopy was performed using a 532 nm laser excitation source and a spectrometer (iHR 550, Horiba) equipped with a grating of 1800 grooves/mm, providing a spectral resolution of approximately 1.3 cm^−1^. The laser power after the 50x objective lens was ∼1 mW to minimize laser‐induced heating. Pelletized polycrystalline CrSbS_3_ was mounted in a sample holder equipped with an open liquid‐nitrogen‐flow cryostat, enabling temperature control from 77 K to room temperature. The selected phonon modes were analyzed by Lorentzian line‐shape fitting to extract their temperature‐dependent Raman shifts.

### 4.8 SHG Measurements

Temperature‐dependent SHG measurements were performed using a Ti:sapphire oscillator with central wavelength at 800 nm. The repetition rate was reduced to 4 MHz by using a pulse picker. After a quarter waveplate, the circularly polarized optical pulses were focused onto the pelletized polycrystalline CrSbS_3_ sample. The sample was mounted in a sample holder equipped with an open liquid‐nitrogen‐flow cryostat, enabling temperature control from 77 K to room temperature. The laser power after the objective lens (54‐17‐29‐UV, Special Optics) was ∼1.5 mW. The SHG photons were collected by the same lens. The fundamental wavelength ∼800 nm was filtered out by using a dichroic beamsplitter and a bandpass filter at 400 nm. A photomultiplier tube (PMT) was used to detect the SHG signals.

## Author Contributions

H.C.W. and H.D.Y. conceived the project. C.N.K. and C.S.L. synthesized the samples. H.C.W., Y.H.L., C.C.T., P.Y.Y., Y.H.C., D.C.K., and J.W.C. performed magnetic, dielectric, polarization, and XAS measurements. C.W.W. carried out the neutron diffraction experiments. D.O. performed synchrotron X‐ray diffraction measurements. T.H.W. and K.H.L. conducted Raman spectroscopy and SHG experiments. H.C.W., Y.H.L., Y.H.C., Y.H.L., C.H.L., D.C.K., A.T., and C.D. assisted with data analysis. H.C.W. supervised the project and wrote the manuscript. H.C.W. and H.D.Y. finalize the manuscript. All authors discussed the results and contributed to the manuscript.

## Conflicts of Interest

The authors declare no conflict of interest.

## Data Availability

The data that support the findings of this study are available from the corresponding author upon reasonable request.
